# Therapist Skepticism and Adaptation to Teletherapy Under Enforced Uptake, With Theoretical Considerations for AI-Mediated Care: Qualitative Study

**DOI:** 10.2196/95939

**Published:** 2026-09-11

**Authors:** Lois Ann Parri, Robin Lau, Karen Addy

**Affiliations:** 1 Department of Psychology University of Cambridge Cambridge United Kingdom; 2 Faculty of Brain Sciences University College London London United Kingdom; 3 School of Sciences, Psychology, Arts and Humanities, Computing, Engineering and Sport Canterbury Christ Church University Canterbury, Kent United Kingdom

**Keywords:** AI, artificial intelligence, clinician attitudes, digital mental health, digital therapeutics, qualitative research, teletherapy, thematic analysis, therapeutic alliance, videotherapy

## Abstract

**Background:**

Mental health care is undergoing rapid digital transformation, with continued development and implementation of digitally delivered interventions. Despite growing evidence for digital mental health interventions, implementation in routine practice has often lagged technological advances. Although teletherapy became more widely accepted during the COVID-19 pandemic, little is known about the psychological processes through which clinicians adapt to disruptive changes in treatment delivery when required to work within a novel medium.

**Objective:**

This study explored how therapists who had not previously adopted teletherapy interpreted and adapted to its enforced uptake during the COVID-19 pandemic, with particular attention to changes in expectations, perceptions of the therapeutic alliance, and professional practice.

**Methods:**

Six UK-based accredited clinical psychologists, none of whom had previously practiced video-based teletherapy, participated in semistructured interviews conducted between May and July 2021, following the rapid shift toward remote delivery. Data were analyzed using reflexive thematic analysis to explore therapists’ experiences of adaptation, belief revision, and therapeutic interactions.

**Results:**

We identified the following four themes: (1) revision of anticipatory beliefs, (2) context-medium differentiation, (3) medium affordances, and (4) relational viability and selective integration. Therapists began practicing teletherapy with concerns about accessibility, safety, therapeutic techniques, and relational quality. With sustained practice, clinicians revised many of their assumptions. Therapists also distinguished difficulties associated with implementation contexts from perceived properties of teletherapy itself. They described adapting clinical procedures and techniques, recognizing both constraints and affordances of the medium, and developing more differentiated views of when teletherapy was appropriate. Clinicians generally found teletherapy to be an acceptable therapeutic medium, but not equivalent to in-person practice.

**Conclusions:**

Adapting to teletherapy involved more than accepting a new tool. It required therapists to reconsider assumptions about therapeutic interaction, safety, and conditions needed to sustain an effective therapeutic relationship. The findings suggest an experience-based professional adaptation process in which initial assumptions became differentiated through sustained use. While these findings support teletherapy uptake specifically, they may also inform how clinicians evaluate other digital mental health technologies, while recognizing that changes to the communication medium are not equivalent to changes in the therapeutic process or agent.

## Introduction

### Digital Transformation in Mental Health Interventions

Mental health interventions have undergone rapid digital development over the past decade. Digital mental health now covers a wide range of technologies, including video-based teletherapy, web- and app-based interventions, digitally adapted treatment tools, and other technology-assisted approaches. Despite accumulating evidence of the effectiveness and promise of these interventions [1-6], implementation in routine clinical practice has often lagged technological advances [7-10]. Commonly reported barriers involve concerns about therapeutic alliance, safety, confidentiality, training, workflow disruption, and organizational readiness [11-13].

The therapeutic alliance is a pan-theoretical concept describing the relational bond and shared agreement on therapeutic goals and tasks between therapist and client [14-16], and is consistently identified as a determinant of treatment outcome [17,18]. Because psychotherapy has conventionally relied on co-present interaction, clinicians have expressed concerns that remote delivery may weaken core relational processes due to reduced nonverbal communication, technological disruption, privacy limitations, and diminished interpersonal presence [19-24]. However, evidence across a range of clinical presentations [25-32] increasingly suggests that a therapeutic alliance can be established and maintained during digital and remote delivery, simply altering therapeutic interactions rather than weakening the relationship [19,20,33-38]. Instead of attempting to replicate in-person therapy online, therapists have described adapting their communication, directed attention, pacing, and relational practices to the remote medium [33,36-38].

### Teletherapy as an Informative Case of Technological Transition

Much of the existing literature on digital mental health implementation has focused on clinicians’ attitudes, perceived barriers, and facilitators of adoption. This work has often drawn on established implementation models such as the technology acceptance model and the unified theory of acceptance and use of technology [39-41]. These approaches help identify factors associated with uptake but are less suited to explaining how clinicians revise assumptions after direct experience with a new therapeutic format.

This distinction matters because clinicians can often act as gatekeepers to implementation, influencing whether technologies are recommended, integrated, or rejected in practice [42,43]. Their evaluations reflect not only evidence about outcomes but also assumptions about what therapy requires, beliefs about relational presence, safety, and what conditions are necessary for therapeutic change. Understanding how such beliefs are maintained, challenged, or revised through experience during technological transitions may therefore explain professional adaptation beyond initial attitudes toward adoption and offer key insights for digital implementation more broadly.

Teletherapy provides a particularly informative case of this professional adaptation process. Unlike most innovations that are adopted gradually and/or selectively, the switch to remote therapy during the COVID-19 pandemic was rapid and, for many clinicians, unavoidable. This created a context in which therapists were required to work within a novel medium, regardless of their prior preferences or confidence. Such circumstances offer a rare opportunity to examine how clinicians interpret experience, revise preceding assumptions, and adapt their practice in response to enforced uptake in contrast to voluntary uptake. Importantly, this context made it possible to study therapists who had not already opted into teletherapy and who, in many cases, may not have adopted it without being required to do so.

Instead of asking only whether teletherapy was effective, the present study examines how therapists interpreted their experiences, how their expectations changed through direct practice, and how they distinguished features of the teletherapeutic medium from features of the wider implementation context. In doing so, the study focuses on how professional assumptions were challenged and revised as therapists adapted to a novel therapeutic medium. Broader implications for other digital mental health technologies are considered as theoretical extensions of these findings in the Discussion section.

### Objectives

This study aimed to examine clinicians’ adaptation to a novel therapeutic medium by qualitatively analyzing therapists’ experiences during early enforced uptake of teletherapy. Specifically, the study sought to (1) explore therapists’ expectations prior to using teletherapy and how these changed through direct clinical experience, (2) examine how the therapeutic alliance was understood and enacted within a remote interactional context, and (3) identify the processes underpinning clinicians’ belief revision during the transition to teletherapy.

## Methods

### Study Design

A qualitative exploratory design investigated clinicians’ experiences of adopting a novel therapeutic medium. The study focused on how therapists interpreted, evaluated, and adapted to teletherapy during its initial implementation during the COVID-19 pandemic. An inductive, experiential approach was chosen to facilitate intensive exploration of perceptions, meaning-making, and belief revision within clinical practice.

### Participants

Participants were recruited via opportunity sampling from May to July 2021 through the “UK Based Clinical Psychology Facebook Group,” an online professional network for accredited clinical psychologists established in 2015. Group administrators verified professional status prior to membership. Exact membership figures at the time of recruitment are not available. With permission from the group administrators, the researchers posted a study advertisement once, inviting eligible clinicians to contact the researcher directly via email. Respondents were screened against the inclusion criteria, which required participants to (1) have delivered therapy via videoconferencing and (2) have had no prior experience conducting remote therapy using video technology before its adoption. Seven individuals responded, were screened, met the inclusion criteria, and provided consent. Six individuals subsequently completed an interview, and 1 did not proceed due to scheduling difficulties. This sampling frame was important because it enabled examination of adaptation among therapists who were not prior adopters of teletherapy. The study, therefore, captured responses to enforced uptake rather than experiences shaped by preexisting enthusiasm for digital practice.

Recruitment continued until an adequate sample size was reached, guided by the principle of information power [44], whereby smaller samples are appropriate when the study aim is focused, the sample is highly specific, the interview dialogue is rich, and the analysis seeks in-depth understanding. The sample consisted of accredited UK clinical psychologists with teletherapy experience but no prior video-based remote therapy experience before COVID-19. Interviews lasted about 45 minutes and yielded relevant, detailed accounts. Given the sample’s specificity, interview depth, and the study’s interpretive aims, LAP and KA deemed the dataset sufficient for in-depth reflexive thematic analysis after 6 interviews.

### Data Collection

Data were collected using semistructured interviews designed to explore expectations, experiences, and changing attitudes toward teletherapy. The topic guide was developed based on the study objectives and gaps in the existing literature. It included 5 professional background questions and 8 open-ended questions with prompts to facilitate elaboration (Multimedia Appendix 1). Questions explored initial expectations, therapeutic alliance, perceived challenges, and interpretations of the implementation context.

Interviews were conducted via Zoom (Zoom Communications, Inc) with audio and video enabled, lasted approximately 45 minutes, and were all conducted in a single session between May and July 2021. Prior to participation, information sheets and consent forms were emailed and signed electronically. Interviews concluded with a debrief, and recordings were stored on an encrypted external drive.

### Data Analysis

Interviews were transcribed verbatim in Microsoft Word and checked twice against the raw recordings for accuracy. Data were analyzed using reflexive thematic analysis following the 6-phase framework by Braun and Clarke [45]. Analysis was carried out using Taguette (version 1.4.1-40-gfea8597) [46].

The first phase consisted of familiarization through repeated reading and listening. Initial codes were generated inductively by identifying pertinent features of the data without applying a preexisting coding framework (Multimedia Appendix 2). Coding prioritized depth of interpretation over frequency. Themes were developed and refined iteratively through repeated review; overlapping themes were combined, conceptually distinct patterns were separated, and theme and subtheme boundaries and labels were revised to ensure internal coherence and distinction between final themes. Refinement was repeated until a stable thematic structure provided a coherent and conceptually meaningful account of the dataset in relation to the research questions. This resulted in 6 rounds of refinement, which was not a predefined number.

The researcher LAP led the analysis, and KA supervised. The research supervisor did not independently code transcripts, and the study did not seek formal inter-coder agreement, consistent with the reflexive thematic analysis approach [45]. Instead, supervision provided a space for critical discussion of coding decisions, alternative interpretations, and the development and refinement of candidate themes. Where different interpretations arose, these were explored through discussion and reconsideration of the relevant extracts and broader dataset, not resolved through consensus coding.

### Reflexivity

Reflexivity was treated as an ongoing analytic resource rather than a source of bias to be eliminated. The researcher LAP had lived experience of in-person and remote therapy (including audio- and video-based delivery) across several of the same therapeutic approaches (cognitive behavioral therapy [CBT], hypnotherapy, and mindfulness). These experiences contributed to a pre-existing view that therapeutic quality may depend more on the therapist’s relational and clinical approach than on the delivery medium itself, given positive and negative encounters in each format. These experiences sensitized the researcher to the possibility that differences associated with teletherapy should not automatically be interpreted as relational deficits, while also creating a potential tendency to underemphasize medium-specific limitations. The researcher had also volunteered for an anxiety helpline, providing prior experience of both the practical limitations of supporting people in distress without physical copresence and the possibility of establishing rapport remotely.

The primary researcher maintained a reflexive journal throughout analysis and discussed positionality during supervision to examine and challenge how their own experiences might shape interpretations, particularly when distinguishing participants’ descriptions of changed therapeutic conditions from claims that those changes represented poorer therapy and when interpreting participants’ views of therapeutic alliance, safety, risk management, and relational presence.

### Ethical Considerations

The study received ethical approval from the Bangor University Research Ethics Committee prior to recruitment (code 2021-16944). Participants provided informed consent and were informed of their right to withdraw at any time. All identifiable information was removed from transcripts, and data were securely stored in accordance with data protection guidelines and Bangor University’s regulations. Participants did not receive financial compensation or reimbursement for taking part in this study.

## Results

### Overview

Six accredited clinical psychologists participated in the interviews. Participants varied in years of clinical practice, therapeutic modalities, and areas of specialism (participant characteristics are provided in Table 1).

Reflexive thematic analysis identified the following four interrelated themes describing clinicians’ experiences of adopting teletherapy: (1) revision of anticipatory beliefs, (2) context-medium differentiation, (3) medium affordances, and (4) relational viability and selective integration. Together, these themes captured a movement from expectation-based skepticism to experience-based reinterpretation. Because participants had not previously chosen to work in teletherapy, the dataset provided a particularly clear view of how clinicians adapted when adoption occurred out of necessity instead of preference. Participants did not describe teletherapy as a degraded version of face-to-face therapy. Rather, they described a distinct therapeutic medium that altered interactional conditions, elicited strong anticipatory concerns, and ultimately required active clinical and relational adaptation. The full table of themes, subthemes, and supporting excerpts is provided in Multimedia Appendix 3.

**Table 1:** Participants’ professional background information.

Participant ID	Years active	Modality and disciplines	Specialism and area of work (if any)
P1	24	CBT^a^ and ACT^b^	No specialist area; works with general adult mental health in individuals and couples
P2	16	CBT, CFT^c^, ACT, and EMDR^d^	Specializes in chronic pain; works with general adult mental health but not personality disorder and severe mental illnesses
P3	25	CFT and CBT	Specializes in psychosis; otherwise treats adult mental health generally
P4	16	CBT, ACT, and EMDR	Specializes in anxiety disorders, mood disorders, trauma, and substance misuse
P5	24	Psychodynamic, EMDR, ACT, EFT^e^, CBT, and mindfulness	No specialist area; works with general adult mental health but not personality disorders
P6	11	Eclectic, primarily CBT	Specializes in anxiety disorders and cognitive rehabilitation

^a^CBT: cognitive behavioral therapy.

^b^ACT: acceptance and commitment therapy.

^c^CFT: compassion-focused therapy.

^d^EMDR: eye movement desensitization and reprocessing.

^e^EFT: emotion-focused therapy.

### Theme 1: Revision of Anticipatory Beliefs

#### Overview

Before and during early use, participants described strong skepticism that teletherapy would be exclusionary, unsafe, and therapeutically limited. These anticipatory beliefs were often revised through direct experience. In many cases, they came to be seen as overstated or incomplete.

#### Anticipated Exclusion

A major concern was that teletherapy would exclude vulnerable clients. Participants worried about a lack of access to devices, internet, money, or digital confidence. P1 referred to “the people it rules out,” while P6 mentioned “people not having the money for the internet and the computer.”

Therapists also worried that more widespread adoption of teletherapy would, as P1 said, “leave people behind.” P1 said that fully replacing face-to-face work could “rule out a good portion of the population,” and P4 reflected that some people may have “lost out” because teletherapy “didn’t feel like it was for them.” These concerns were framed as logistical issues beyond questions of equity and access.

At the same time, participants’ experiences often complicated these assumptions. Several described encountering a wider and more digitally capable client group than expected. P2 reflected on their “own prejudices,” having assumed teletherapy would mainly attract younger people, but instead found “a wide range of difficulties and a wide range of areas and ages.” P3 described an older client, “nearly seventy,” who initially needed help setting up Zoom but later used it independently. P6 similarly reported that clients rarely objected and often said, “this works okay, I quite like this.” These did not eliminate concerns about exclusion, but they suggested that therapists’ initial assumptions about who teletherapy would and would not work for were often too narrow.

#### Anticipated Risk

Risk was another major concern. Participants worried that teletherapy would reduce their ability to manage distress, respond effectively in crisis situations, or intervene when clients became overwhelmed. P5 described “one of my real fears” as the possibility that a client might go “right out their window of tolerance” while the therapist remained “powerless” because the client was “somewhere else.” These concerns suggested that initial skepticism about teletherapy was shaped partly by concerns about safety, containment, and the limits of remote intervention.

At the same time, these concerns were often moderated through practice. Instead of concluding that teletherapy was inherently unsafe, therapists described learning to manage some risks through procedural adaptation. P4 explained the importance of measures such as obtaining “an emergency contact number” in case a client “just disappeared” from an online session, and described becoming more used to “thinking about the safety stuff” over time. They also reflected that what had initially “felt, risky” became more manageable through “unpicking what that riskiness is,” “quantifying that,” and “putting in extra safety measures.” These suggested that anticipated risk was not removed, but became more differentiated and manageable through experience.

#### Anticipated Therapeutic Insufficiency

Participants also anticipated that teletherapy would be therapeutically insufficient for some forms of work. These concerns centered on modality fit, practical therapeutic tasks, and higher-risk clinical situations. P1 stated that they had not “felt comfortable” doing eye movement desensitization and reprocessing (EMDR) online. P6 similarly suggested that some tasks and modalities “doesn’t work as well” remotely, especially when they require more practical or hands-on elements. These anticipations suggested that early skepticism was also shaped by doubts about whether teletherapy could adequately support certain kinds of therapeutic work.

At the same time, direct experience often challenged these expectations. P1 reflected that trauma-focused CBT had “worked ok” online and wondered whether they were “just finding barriers” for themselves. Therapists also described adapting methods, not abandoning them entirely. P3 noted that chair work was harder online, but explained that therapists were finding ways to modify it, for example by using “different hats or a different colour scarf.” Similarly, P5 initially struggled to believe that adapted EMDR techniques would work, but after experiencing tapping as an alternative to on-screen visual tracking, said it was “really so super effective” and “really surprised” them. Together, these accounts suggested that anticipated therapeutic insufficiency was often revised through practice and adaptation.

### Theme 2: Context-Medium Differentiation

#### Overview

Participants described being introduced to teletherapy alongside a number of substantial changes in working conditions, therapeutic settings, household routines, and wider social circumstances. In some accounts, participants explicitly distinguished difficulties they attributed to the implementation context (the COVID-19 pandemic or working from home) rather than the medium itself. The study cannot independently determine the medium vs the context’s objective causal contribution to these difficulties. Instead, this theme captures how participants situated and, in some cases, reinterpreted their teletherapy experiences within the wider implementation context.

#### Home-Working and Boundary Disruption

Several participants described disrupted professional boundaries alongside the move to teletherapy when working from home. P1 referred to the “blurring” of work and relaxation time, while P2 emphasized the challenge of “letting someone into your home” in contrast to a “normal therapy space.” These participants did not necessarily identify a single cause for these experiences. Analytically, their opinions show that the transition to teletherapy coincided with a major change in the domestic and professional conditions of therapy.

#### Therapeutic Setting Changes

Participants also reflected on how the therapeutic setting had changed. P2 initially reported the significance of consistency, but then said in some cases, the changed setting appeared to humanize the therapist. P2 suggested that clients sometimes “quite like” seeing the therapist as “a person” during minor domestic interruptions, such as the doorbell or a dog entering the room.

At the same time, therapists described efforts to recreate a therapeutic environment within the home context. P3 explained that therapists and clients had to “create a therapy room here online” by guaranteeing privacy, appropriate lighting, and reduced distractions. These accounts illustrate how participants experienced the teletherapeutic medium together with changes in the therapeutic setting. Where participants did not directly report the source of these effects, they are treated as contextual information, not evidence of causality.

#### Privacy and Household Interruption

Privacy and household interruption were recurring challenges. P4 described a client with school-aged children having “no way” to obtain the physical or mental space needed for sessions when schools were closed. P6 similarly acknowledged that interruptions were common in households with children, although they also noted that “being able to access therapy even though you’ve got disruption is better than having no therapy at all.”

These examples could easily be interpreted as problems from the medium or the context. These reports show how difficult it is to separate features of teletherapy from the conditions under which it was delivered during lockdown.

#### Explicit Differentiation of Pandemic and Medium

At times, participants explicitly differentiated difficulties associated with the broader pandemic context from those they had initially attributed to teletherapy. P1 referred to some therapy-related difficulties as “COVID barriers rather than online therapy barriers,” particularly when clients lacked opportunities to engage in real-life behavioral tasks. P6 similarly distinguished the isolation of home working from the teletherapy medium itself, describing the loss of office-based collegial support as “the pandemic situation really.”

Participants also observed that altered face-to-face work under wider pandemic constraints could itself be less relational than video contact. P1 remarked that socially distanced face-to-face work involving “masks and distance” had “felt harder, in terms of therapeutic alliance, than on video, interestingly.” Even some apparently positive outcomes were linked to the surrounding context rather than the medium itself. For example, P4 noted that lockdown had “suited” some anxious clients by reducing external demands.

Together, these interpretations provide the clearest evidence that participants can differentiate the teletherapeutic medium from the circumstances surrounding its implementation. They indicate retrospective reinterpretation of experience, but they do not definitively establish which factors caused the difficulties described. The pandemic provided a particularly vivid example of this process.

### Theme 3: Medium Affordances

#### Overview

Participants described teletherapy as changing the practical and interactional conditions of therapy. They experienced these changes as both constraining and enabling.

#### Digital Resources and Technical Mediation

Participants reported that teletherapy changed how they used therapeutic materials. Several initially found it difficult to share diagrams, formulations, or written resources in the same spontaneous way as in person. P1 noted that “the sharing of materials initially was quite difficult,” while P2 described “not being able to just write something down on a piece of paper” or “draw a formulation” during the session. However, participants also described learning to use digital functions more flexibly over time. P2 reflected that there were conveniences, such as “quickly bring it up on my screen” when sharing a video or resource.

At the same time, therapists described technical disruption as a medium-specific constraint. Connectivity problems were particularly problematic during emotionally intense moments. As P6 explained, when a client is upset and “they freeze” due to poor video connectivity, the interaction is broken and “that is awful.”

#### Reconfigured Communicative and Embodied Cues

Participants consistently described teletherapy as changing what could be perceived and expressed interpersonally. Some aspects of embodied response were reduced. P2 stated that it makes “the empathy a little harder, the lean in isn’t possible,” and P3 noted that “touch is missing,” including gestures such as a handshake or hand on an arm.

Participants also described concerns about missing information that would normally be visible in person. P6 referred to the possibility that a client “might be doing something off-screen” that would be an important clinical indicator. However, this was not only described as a loss. P5 noted that there was sometimes “less to be missed” in clients’ facial expressions because of the “close-up view.” These views suggested that communicative cues were not just diminished but reconfigured.

#### Intensified Attentional Demands

All participants reported that teletherapy was more cognitively demanding than face-to-face work. They reported sustained visual focus, lessened environmental distraction, and heightened attentional effort. P6 explained that online interaction required therapists to be “focused on the people you’re talking to more,” which made it “more exhausting.” P4 similarly described the experience as “more intense,” and suggested that this level of focused attention could also feel uncomfortable for clients.

P1 noted that teletherapy could be “quite tiring,” particularly for those unfamiliar with the format. Although participants could not always fully articulate the mechanism underlying this fatigue, they consistently described teletherapy as changing the attentional texture of sessions.

#### Accessibility, Flexibility, and Autonomy

Regardless of these challenges, participants consistently emphasized greater accessibility and flexibility. Teletherapy reduced travel demands, widened geographical reach, and made attendance easier to fit around work and family life. P1 referred to the “breakdown of the barriers of location,” while P3 stated that teletherapy “makes it more accessible for people with mobility issues or anxiety issues.”

P4 described the benefits in detail, noting that therapy that previously required “two or even three hours,” including travel, instead “becomes an hour a week.” Participants also highlighted greater client choice and autonomy. P4 noted that clients now had a wider geographical pool of therapists, while P6 described arranging sessions so that clients initiated contact themselves, giving them “that sense of power over a session.”

#### Differential Client-Medium Fit

Participants further observed that teletherapy appeared to suit some clients especially well. P1 described seeing more “IT people” and more “university students” who benefited from continuity as they moved between home and university. P6 also noted that some people with “autistic spectrum disorders” appeared to “find it [therapy] easier online.” These accounts suggested that the fit between client and medium was variable rather than uniform.

### Theme 4: Relational Viability and Selective Integration

#### Overview

The final theme captured how therapists increasingly came to view teletherapy as a viable, though distinct, relational format. Rather than describing teletherapy as inferior to face-to-face work, participants reflected on whether meaningful therapeutic relationships could be established online and, if so, under what conditions. These suggestions indicated that teletherapy was often seen as workable and beneficial, but not in a uniform or universal way.

#### Alliance Preserved But Reconfigured

Across interviews, therapists came to see the therapeutic alliance as achievable online, although not identical to face-to-face work. P1 reflected that although alliance formation had initially seemed likely to be “more difficult,” it later felt as though it “manifests itself anyway, without you having to be in the same room.” P5 similarly stated that “the way that I respond to people is based on their response to me,” and that they did not think there was “anything about doing it through Zoom” that had really interfered with developing a relationship with clients. P5 also described having been “quite anxious” at first about not being able to connect with or read clients properly, but later felt they had been “proved completely wrong.”

Other participants emphasized difference rather than equivalence. P3 described online therapy as “a different kind of relationship,” that there are “good relationships being built,” and “it still feels trusting.” P6 was more cautious, suggesting that in-person work could allow “a more close relationship.” Overall, however, participants did not describe the alliance as lost. Rather, they described it as altered in texture and cues, but still possible.

#### Evaluating Fit and Sustaining Integration

Participants also increasingly evaluated teletherapy as effective and worth retaining, although in selective rather than universal ways. P2 described a broader shift in attitude, noting that “we all thought” online therapy would be “too difficult,” but that it had been “a lot better” than expected. At the same time, therapists stressed that its value depended on fit. P1 noted that “every client” and “every therapist” would experience it differently, suggesting that teletherapy should not be treated as equally suitable in all cases.

This more differentiated evaluation was reflected in how therapists imagined teletherapy’s longer-term place in practice. P4 planned to maintain both an in-person and an online clinic because the 2 formats could “run nicely side by side,” and emphasized the importance of giving clients a choice. P6 similarly felt teletherapy could improve access for many people, but that “not all types” of therapy should be replaced. Some participants were especially positive, with P3 saying they were “considering staying online” and P5 stating that they would “prefer...to continue to make it work.” Overall, participants described teletherapy as workable, beneficial, and, in many cases, sustainable beyond the immediate circumstances in which it had been adopted. As P3 summarized, “we can develop a really good therapeutic relationship, and people are getting better. So, it’s working.”

## Discussion

### Principal Findings

This study explored therapists’ experiences of adopting video-based teletherapy and how their expectations, attributions, and understanding of therapeutic processes changed through direct use. In line with earlier research showing that remote therapy can be clinically effective and that therapeutic alliance can be maintained online [32,34,35,38], participants in the present study described teletherapy as both workable and, in some circumstances, advantageous. However, the present findings extend beyond the conclusion that teletherapy can work. They suggest that uptake involved a wider process of professional adaptation in which therapists reconsidered assumptions about what is required and learned to practice within a novel therapeutic medium.

Across interviews, participants described entering teletherapy with strong anticipatory apprehensions about safety, relational depth, accessibility, and modality fit. These concerns were not necessarily unfounded, but they were assumptions based on limited prior experience. Nevertheless, many were reinterpreted after gaining direct experience over time. Therapists found that some clients were more comfortable with technology than anticipated, that risk could often be managed by alternative procedures, and that therapeutic techniques could be effectively adapted. They also realized that the therapeutic alliance could still be established online, even if some building blocks differed from face-to-face practice.

Participants also went beyond simply comparing treatment outcomes across both formats and interpreted the meaning of their experiences, revising their explanations over time. Some of the early difficulties were initially attributed to properties of the teletherapeutic format. With continued experience, many of these assumptions were revised, and participants later reinterpreted them as features of home working, household interruption, pandemic restrictions, or the wider implementation context and proceeded to recognize previously unanticipated affordances and adapt their clinical practices accordingly. Their eventual evaluations were more nuanced: teletherapy was not a degraded substitute for in-person work but a distinct medium that altered the conditions of therapeutic interaction in both constraining and enabling ways.

Overall, the findings suggest that clinicians’ adoption involved conceptual and professional learning, as well as technological uptake. Therapists developed new safety procedures, altered how they shared materials, adapted specific therapeutic exercises, and learned to attend to relational cues in the online environment. Early resistance to teletherapy was grounded less in direct evidence of failure and more in assumptions about irreplicable therapeutic processes, particularly concerns about co-present interaction. When those assumptions were contradicted by experience, participants also revised their assumptions about what was needed for alliance, safety, and therapeutic effectiveness.

### A Process of Professional Adaptation

Across these 4 themes, a process of adaptation became apparent. Clinicians moved from anticipatory beliefs characterized by concerns grounded largely in speculative assumptions to an early period in which features of the implementation context were not always clearly differentiated from teletherapy as a medium, and ultimately to experience-based belief revision through sustained interaction with the medium. Direct experience with teletherapy is what allowed participants to recognize its constraints and affordances, revise earlier assumptions, and accept the new therapeutic context.

**Figure 1:**
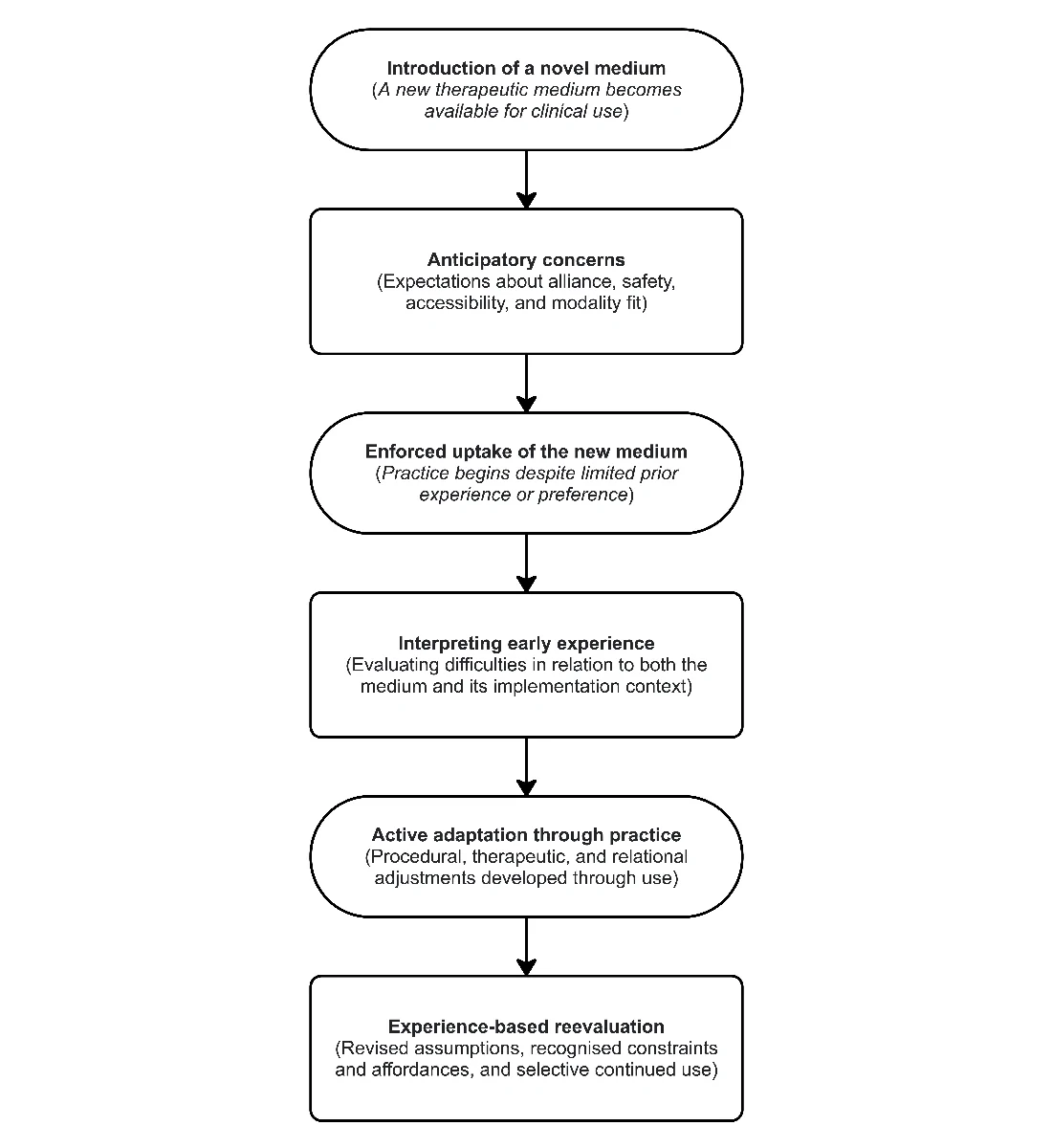
Model of clinician adaptation to a novel therapeutic medium. Rectangular boxes represent internal processes, and rounded boxes represent external events or actions. The model synthesizes processes across the 4 themes and depicts a conceptual, rather than fixed or universally linear, sequence of adaptation.

This process is represented in Figure 1 as a model moving from anticipatory beliefs, through implementation and context-medium differentiation, toward sustained interaction and experience-based belief revision. This process should not be interpreted as uniform progression from skepticism to endorsement. Participants retained concerns about client access, privacy, risk, fatigue, modality fit, and the loss or alteration of certain embodied cues. Their final evaluations were generally selective, not unconditional. Teletherapy was viewed as viable by all for many clients and purposes, but not necessarily as the preferred or most appropriate format for every situation.

This model helps explain why teletherapy uptake has historically lagged evidence of its effectiveness. Clinicians were not evaluating teletherapy only in terms of treatment outcomes. They were also anticipating the disruption the medium change might pose to core therapeutic principles, including empathy, alliance, safety, and presence. Since these issues were based on clinicians’ therapeutic understanding and practice to date, evidence of efficacy alone may not have been sufficient to resolve concerns. Direct and sustained interaction with the new medium, albeit enforced during a time when face-to-face therapy was not permitted, provided the opportunity to challenge these assumptions, identify which concerns were substantiated, develop adaptations as required, and revise their evaluations.

The proposed model is intended to complement rather than replace established models of technology acceptance. The technology acceptance model emphasizes perceived usefulness and perceived ease of use as determinants of technology acceptance [39,40], while the unified theory of acceptance and use of technology extends this perspective by incorporating performance expectancy, effort expectancy, social influence, facilitating conditions, and the moderating roles of factors including experience and voluntariness [41]. These frameworks are particularly useful for explaining variation in intention to adopt and subsequent technology use. The present findings address a somewhat different process: clinicians began using teletherapy despite limited prior experience or preference because external circumstances required its uptake. In this context, use unusually preceded positive acceptance.

The present model therefore foregrounds what happened once enforced uptake began. Anticipatory concerns were confronted with direct clinical experience; clinicians interpreted early difficulties in relation to both the medium and its implementation context, developed procedural, therapeutic, and relational adaptations through practice, and subsequently reevaluated some earlier assumptions while retaining others. Its proposed contribution is therefore not a competing general model of technology acceptance, but a process-oriented account of how professional beliefs and practices may be revised through sustained experience when uptake precedes voluntary acceptance.

Three features of this process are particularly important.

First, anticipatory concerns influence initial evaluations. Participants described numerous expectations that emerged before meaningful practice, including concerns about exclusion, relational insufficiency, and the inability to deliver more complex forms of therapy online. These expectations were not irrational, but many were later moderated or revised through experience.

Second, differentiation between the therapeutic medium and its implementation context shaped clinicians’ judgments. Participants often encountered genuine difficulties, but the cause of those difficulties was not always clear. Teletherapy was introduced during an unusually disruptive period in which remote clinical work coincided with home working, pandemic restrictions, household interruptions, and broader social disruption. Some participants explicitly distinguished difficulties they later associated with these wider conditions from those they initially assumed were properties of teletherapy itself. Other contextual distinctions were not stated directly by participants, but identified through the present analysis. The study cannot establish the objective causal source of these experiences or cleanly separate effects of teletherapy from effects of the pandemic. What the accounts do show is that clinicians’ evaluations of the medium were formed within a particular implementation context and were, in some cases, subsequently reinterpreted as participants gained direct experience. This highlights the importance of considering implementation context when examining professional adaptation to new therapeutic technologies to avoid distorting evaluations about a technology’s acceptability or effectiveness and needlessly slowing implementation.

Third, adaptation was active, not passive. This component of the model draws across the themes rather than corresponding to a single theme. In theme 1, participants described developing practical and clinical adaptations in response to concerns that had accompanied early teletherapy use, including additional emergency-contact procedures, adapting chair work using visual props, and modifying EMDR techniques. Theme 3 further identified medium-specific constraints and affordances that required changes in how therapists shared materials, directed attention, and communicated, while Theme 4 showed how relational practice and longer-term use were selectively adjusted to the medium. Active adaptation therefore occurred alongside sustained experience: participants did not only become more tolerant of teletherapy, but modified their procedures, techniques, and relational practices as they learned what the medium required.

The findings reframe adoption as a process of professional adjustment, not simple acceptance of a new tool. Successful implementation requires more than just technical access or platform availability. It requires clinicians to reinterpret what therapeutic presence looks like, how alliance is formed, and how safety can be maintained when the mode of communication changes.

### Implications for Teletherapy Training and Implementation

These findings suggest that barriers to adoption are not solely technical or organizational. They may also be interpretive. Clinicians’ responses to a new therapeutic medium are shaped partly by their existing beliefs about how therapy works and which conditions are essential for effective practice. Training and implementation efforts may therefore benefit from explicitly addressing how clinicians make sense of new therapeutic formats, instead of focusing on technological competence.

First, training may help clinicians distinguish medium-related effects from contextual effects when evaluating a new intervention. For example, privacy problems arising because family members are also at home and professional isolation associated with home working should not be automatically attributed to inherent properties of teletherapy. Training should acknowledge and address genuine medium-specific challenges, such as connectivity failures, changes in embodied communication, and screen fatigue.

Second, training should aim to identify which therapeutic mechanisms are more reliant on human engagement and which are more robust to format changes. Then, providing structured opportunities to learn and rehearse such adaptations will begin to provide direct experience, which in turn might reduce reliance on speculation and sooner break down unsubstantiated assumptions. This could also manage expectations that every intervention can be transferred online without adaptation.

Third, supervised exposure to remote practice may reduce speculative concerns by allowing clinicians to test assumptions through experience rather than through prediction alone. The present findings suggest that most concerns were only revised after working directly with a range of clients and encountering firsthand what does and does not work. Supervision with a focus on the new teletherapeutic medium could provide a setting that facilitates reflective awareness and challenges assumption-based reasoning.

Finally, implementation efforts should retain a focus on differential fit and avoid aiming to fully replace one with another. Participants emphasized variation in teletherapy appropriateness between clients, therapists, circumstances, and therapeutic needs. Exposure to teletherapy may reduce excessive skepticism, but training should also encourage critical evaluation so that positive adaptation does not become uncritical generalization. The goal is not to promote or resist digital mental health technologies in principle, but to improve the accuracy with which they are evaluated.

### Conjectural Implications for Broader Digital Mental Health Services

Although these outcomes arise specifically from teletherapy, their relevance may go beyond remote delivery, as clinicians often interpret new technologies through prior experiences of adaptation. Different forms of digital mental health services alter different aspects of care. Teletherapy changes the communication channel while retaining a human therapist. Structured digital interventions may reorganize how therapeutic tasks are delivered. AI-mediated systems may alter or partially automate the therapeutic agent itself. These are not interchangeable changes, even when they are grouped together under the umbrella of digital mental health.

One way to interpret the present findings is through schema formation. Schema theory proposes that people use organized knowledge structures to interpret incomplete and unfamiliar information, and research on analogical transfer suggests that experience with one class of cases can generate a broader schema that is then applied to later cases [47,48]. In this context, successful adaptation to teletherapy could help clinicians build an internal model of what “digital therapy” is and how it works. That model may then shape how subsequent technologies are interpreted and judged.

This may be beneficial given that it reduces assumption-based resistance to innovation. However, it may also create a risk of losing important nuance if later technologies are interpreted through a schema formed primarily around teletherapy. In other words, clinicians may carry forward a mental model derived from a change in communication medium and apply it too broadly to systems that change something more fundamental. The present data do not show that clinicians have already done this with AI-mediated therapy. Rather, they suggest a plausible implementation risk: later technologies may be assimilated into an existing “digital therapy” schema when they may instead require a more differentiated evaluation. In other words, successful digitization of the medium should not be assumed to imply that digitization or automation of the therapeutic agent will preserve the same mechanisms of care.

This point is especially relevant given the growing interest in AI-based conversational and large language model systems in mental health care. Recent work argues both that AI-assisted decision-making depends on users having accurate mental models of what AI systems can and cannot do, and that large language model–based behavioral health care represents a distinct and high-stakes development rather than a simple extension of existing teletherapy [49,50]. The challenge is therefore not only whether clinicians accept new technologies, but whether they evaluate them with sufficient conceptual precision.

### Strengths and Limitations

This study provides an in-depth qualitative account of clinician adaptation during a naturalistic technological transition. By capturing the views of therapists who had not previously voluntarily delivered teletherapy and who, in many cases, may not have moved to this mode of working without being required to do so, the study setting provides a distinctive opportunity to study the psychological adjustment involved when clinicians are required to work with a novel therapeutic medium. This process may differ from adaptation among early adopters or clinicians already positively oriented toward technological change, whose uptake is already likely to be voluntary and whose initial observations may therefore differ from those observed here. This offered a useful counterpoint to studies of voluntary uptake, where attitudes and experiences may be shaped by pre-existing openness to digital practice. The varied professional backgrounds further strengthened the variety of perspectives.

However, several limitations should be considered. The sample was small and professionally homogeneous, comprising 6 UK-based accredited clinical psychologists. Client perspectives, which could have offered complementary insights, were not represented. Although the focused sample supported in-depth examination of the research questions, the findings may not reflect the experiences of other mental health professionals, service settings, or clinicians with different levels of prior engagement with digital practice. Participants were also recruited through a single online professional group, which may have preferentially reached clinicians who were more active in professional networks, potentially introducing recruitment bias. Accounts were retrospective and therefore may have been shaped by recall and retrospective reinterpretation. In addition, focusing on teletherapy introduced during the exceptional circumstances of the COVID-19 pandemic further limits transferability to other implementation contexts. Adaptation under elective or routine teletherapy implementation may therefore differ from the process described here. The findings reflect a specific implementation context and should be interpreted as theory-generating, not generalizable estimates. Future research should explore whether these results are evident across more diverse professional groups, clinical settings, and implementation contexts.

### Conclusions

The transition to teletherapy revealed a recurring process of professional adaptation involving anticipatory concern, differentiation between the therapeutic medium and its implementation context, and experience-based belief revision. Therapists did not only learn to use a new technology; they also reconsidered assumptions about the nature of therapeutic interaction, the conditions required for alliance, and the practical management of risk. This process supported teletherapy uptake and helped explain why remote therapy came to be seen as a viable mode of care.

At the same time, this study suggests that learning from one technological transition may influence responses to future ones. As mental health care continues to diversify digitally, accurate evaluation will depend on distinguishing which aspects of therapy are being changed: the communication medium, the treatment process, or the therapeutic agent itself.

Understanding clinician adaptation is therefore relevant not only to teletherapy but to digital mental health implementation more broadly. The challenge for future practice is not adopting or rejecting new technologies. It is learning from prior transitions without drawing overly broad conclusions.

## Appendix

Multimedia Appendix 1 Interview questions.

Multimedia Appendix 2 Initial codes.

Multimedia Appendix 3 Themes, subthemes, and supporting excerpts.

Multimedia Appendix 4 Transcript of author's generative AI use.

## Abbreviations

CBT: cognitive behavioral therapy
EMDR: eye movement desensitization and reprocessing

## Data Availability

The datasets generated and analyzed during the current study are not publicly available due to confidentiality but are available from the corresponding author on reasonable request.
