# Supplementary material for: Therapist Skepticism and Adaptation to Teletherapy Under Enforced Uptake, With Theoretical Considerations for AI-Mediated Care: Qualitative Study
Source: JMIR Form Res. 2026 Sep 11;10:e95939. doi: 10.2196/95939 (PMC13615378; doi:10.2196/95939)
Supplement: Supplementary file 1 [file formative_v10i1e95939_app4.pdf]

This is a copy of a chat between Claude and Lois. Content may include unverified or unsafe content that do not represent the views of Anthropic. Shared snapshot may contain attachments and data not displayed here.

Report

Files hidden in shared chats

I have been revising this manuscript along with my co-authors for resubmission. Proofread to check for any typos or inconsistencies. I am attaching my response to the review comments for context.

Ran 19 commands, viewed 10 files

Good, quotes are consistently curly. Let me check apostrophe consistency and a few remaining sections I haven't fully verified (limitations/references cross-check with response letter comments 112-283 that were truncated).

Ran 3 commands, viewed a file

Response letter looks clean of these basic issues. Now let me verify the "Work(if any)" typo directly in the clean docx (not just tracked) to confirm it's present in the version being resubmitted, and do a final check on a couple of other small things.

Ran a command

Good — I've read through the full manuscript (clean version), spot-checked it against the tracked-changes file and your response-to-reviewers letter for consistency, and verified all in-text citations resolve correctly against the reference list. Here's what I found:

Likely typos to fix

1. **Table 1 header** — "Specialism and Area of Work**(if any)**" is missing a space: should read "Work (if any)".
2. **Theme 2, "Therapeutic setting changes" paragraph** — stray double period: "...they are treated as contextual information, not evidence of causality**..**" should end with a single period.
3. **Theme 1, "Anticipated Risk" paragraph** — one quoted phrase has what looks like a stray comma: "what had initially *'felt, risky'* became more manageable..." — worth checking against the interview transcript, since if it's a verbatim quote the comma may reflect a pause in speech, but as written it reads like a typo (*"felt risky"*).

Consistency point worth a look (not necessarily wrong)

4. The Abstract's Results sentence uses the phrase "difficulties associated with **socio-environmental contexts**," but this term doesn't appear anywhere else in the manuscript — the body consistently uses "**implementation context**" instead (including in the Theme 2 heading/discussion). Since your response letter documents how carefully the editor scrutinized Theme 2's terminology, you may want to align the abstract's wording with "implementation context" for consistency.

Everything else checked out cleanly:

- All 49 references are cited in the text and none are orphaned; the reference corrections described in your response letter (refs 42, 46, 47) are correctly reflected in the current manuscript.
- The revised title ("...with Theoretical Considerations for AI-Mediated Care") matches what you describe having changed for Editor Comment 2/Reviewer BX Comment 3.
- Terminology from your response letter (Theme 2 renamed to "Context-medium differentiation," structured abstract, AI framed as conjectural, etc.) is consistently reflected throughout.
- British spelling is used consistently (no stray American spellings crept in outside quoted reference titles).
- Curly quotes/apostrophes are used consistently throughout (no stray straight quotes).
- No repeated words, and the tracked-changes file's accepted content matches the clean file (aside from the expected edit history).

Want me to make these edits directly in the docx for you?
