# Supplementary material for: Therapist Skepticism and Adaptation to Teletherapy Under Enforced Uptake, With Theoretical Considerations for AI-Mediated Care: Qualitative Study
Source: JMIR Form Res. 2026 Sep 11;10:e95939. doi: 10.2196/95939 (PMC13615378; doi:10.2196/95939)
Supplement: Supplementary file 2 [file formative_v10i1e95939_app1.docx]

**Multimedia Appendix 1. Interview Questions**

Exploring the Impact of Treatment In-Person or by Teletherapy on the
Therapeutic Alliance during Covid-19

Semi-Structured Topic Guide

Demographic/Background Questions

1) How long have you been a practicing therapist?
2) What are the therapy modalities and disciplines do you use? e.g. CBT, Hypnotherapy,
   Person-Centred, Mindfulness, Eclectic
3) What type of clients do you treat? E.g. adults or children, groups or individuals?
4) Do you specialize in any type of mental health issue? E.g. mood disorders, eating
   disorders, anxiety disorders?
5) What working sector do you belong to? E.g. private, public or third?

Main Questions

1) Is teletherapy a feasible alternative to in person treatment?
2) What are the main differences of in person treatment and teletherapy?
3) Do you feel a barrier by not treating in person?
4) Have clients been open to the change?
5) Is there a difference to the therapeutic alliance when conducting treatment in
   person or by teletherapy?
6) Are there differences to the therapeutic alliance when conducting treatment in
   person during the covid-19 pandemic compared to before the pandemic?
7) What do you think are the benefits / downsides of teletherapy?
8) Do you think teletherapy could be a feasible alternative to replace in person
   treatment?
