# Supplementary material for: Therapist Skepticism and Adaptation to Teletherapy Under Enforced Uptake, With Theoretical Considerations for AI-Mediated Care: Qualitative Study
Source: JMIR Form Res. 2026 Sep 11;10:e95939. doi: 10.2196/95939 (PMC13615378; doi:10.2196/95939)
Supplement: Supplementary file 3 [file formative_v10i1e95939_app2.docx]

**Multimedia Appendix 2. Initial Codes and Frequency**

| **CODE** | **FREQUENCY** |
| --- | --- |
| Autism Spectrum Disorder Population | 1 |
| Alternative Not Replacement | 8 |
| Anxiety Population | 3 |
| Audio Only Teletherapy | 2 |
| Benefit of Replacing | 1 |
| Blend of Both in Future | 7 |
| COVID Issue | 3 |
| COVID In-person Would be Worse | 7 |
| Client Engagement + | 9 |
| Client Engagement - | 5 |
| Client Safety Worries | 10 |
| Clients Enjoy Digital | 9 |
| Client’s Prejudices | 7 |
| Convenient + | 5 |
| Cost Effective + | 4 |
| Digital Resources | 7 |
| Disconnect in Experience | 4 |
| EMDR + | 5 |
| EMDR - | 6 |
| Employed Population | 1 |
| Environmentally Friendly + | 1 |
| Flexibility+ | 10 |
| Geographical Flexibility+ | 12 |
| Humanises Therapist Work From Home | 1 |
| IT Population | 1 |
| In-Person Presence | 29 |
| Individual Differences | 18 |
| Interruptions + | 2 |
| Lack of Physical Resources | 8 |
| Lack of Privacy at Home | 6 |
| Language ACC+ | 1 |
| Maintaining Consistency Worm From Home | 6 |
| Maintaining Personal Boundaries Work From Home | 4 |
| Missing Social Cues | 25 |
| Money Accessibility- | 1 |
| More Attention + | 1 |
| More Choice + | 4 |
| More Control + | 6 |
| NHS and Bigger Organisations | 2 |
| Neuropsychology Evaluations - | 1 |
| New Challenges | 30 |
| No Change | 2 |
| Obligation Flexibility+ | 1 |
| Older Population | 2 |
| Online More Intense | 7 |
| Online is Tiring | 4 |
| Online is a Success Because People Are Desperate | 2 |
| Open to Move Because No Choice | 3 |
| Overcome-able Challenges | 19 |
| Parent Population | 1 |
| People Missing Out | 4 |
| People Who Were Previously Reluctant are No Longer Reluctant | 3 |
| Physical Ability Accessibility+ | 5 |
| Physical Ability Accessibility- | 4 |
| Physical Hospitality | 1 |
| Psychodynamic - | 1 |
| See more Clients | 1 |
| Sense of Distance - | 1 |
| Student Population | 2 |
| Therapeutic Alliance | 17 |
| Therapeutic Alliance Develops the Same | 9 |
| Therapeutic Alliance is Different | 5 |
| Teletherapy Requires More Mental Effort | 1 |
| Teletherapy Works | 22 |
| Teletherapy can Replace In-person | 6 |
| Teletherapy is More Difficult | 1 |
| Technology | 30 |
| Technology Accessibility- | 8 |
| Technology Issues | 1 |
| Therapeutic Space | 10 |
| Therapist Pre-Judgement has Changed | 14 |
| Therapy Modality Not Working | 15 |
| Therapy Techniques | 13 |
| Time Flexibility+ | 13 |
| Trauma Issues | 5 |
| Travel Flexibility+ | 14 |
| Travel Flexibility- | 2 |
| Unfamiliar with Technology | 6 |
| Unrelated to Online | 2 |
| Varied Population | 1 |
