# Supplementary material for: Therapist Skepticism and Adaptation to Teletherapy Under Enforced Uptake, With Theoretical Considerations for AI-Mediated Care: Qualitative Study
Source: JMIR Form Res. 2026 Sep 11;10:e95939. doi: 10.2196/95939 (PMC13615378; doi:10.2196/95939)
Supplement: Supplementary file 4 [file formative_v10i1e95939_app3.docx]

**Multimedia Appendix 3. Full Table of Themes, Subthemes, and Supporting Excerpts**

| **THEME** | **THEME DESCRIPTION** | **SUBTHEME** | **SUBTHEME DESCRIPTION** | **SUPPORTING EXCERPTS** |
| --- | --- | --- | --- | --- |
| 1. Revision of Anticipatory Beliefs | Before or during early teletherapy use, therapists anticipated exclusion, risk, and therapeutic insufficiency. These expectations were often revised through direct experience. | 1.1. Anticipated Exclusion | Therapists expected teletherapy to exclude vulnerable clients and those lacking access, money, or digital skills, but experience often challenged these assumptions. | P1: “Perhaps the people it rules out, because they don’t have access to the, the, technologies or, erm, or they have some kind of difficulty which makes accessibility not practical, not possible…”  P6: “Erm, the other downside, accessibility in term of, you know, people not having the money for the internet and the computer and all that kind of stuff”  P1: “Erm, but I think it’s going to be, if you completely replace it you rule out a good portion of the population, potentially some more vulnerable people within the population. So I think, um, if I, I wouldn’t entirely replace it, erm. But I think, yeah, the risks are that you’d lose people and leave people behind that really do need help, so, yeah.”  P4: “Erm, but I do wonder how many people, erm, lost out actually, cause the, they couldn’t or they didn’t feel like it was for them.”  P2: “My own prejudices, I had assumed that that we would be capturing a young group of people. But that has not been the case, there’s been a wide range of difficulties and a wide range of areas and ages.”  P3: “Like, a week or two before lockdown. And I think people were happy with that, having considered their health and wellbeing, they were really open to moving online, and new people, I think people are more used to being online. So, they’re like “oh online therapy, that’s the thing, that’s the way it is”. Even my older lady whose nearly seventy, she’s had to get help from her daughter to set it up and learn how to use zoom, but now she’s fine with using it by herself so yeah.”  P6: “Again yes, but because there’s no alternative, I suppose, as opposed-You know I haven’t had a client that’s complained to me about it, what I’ve had is clients saying, “do you know what, this works okay, I quite like this”, you know.” |
|  |  | 1.2. Anticipated risk | Therapists worried teletherapy would be unsafe in higher risk or emotionally intense situations, though practice showed some of these risks could be managed through adaptation. | P5: “So, one of my real fears is that somebody would sort of go right out their window of tolerance and not be able to deal with what’s in their mind, and I’m powerless at doing anything about it because they’re you now, they’re somewhere else.”  P4: “Erm, so making some of the adaptations I was asking for, it’s so helpful to be linked to kind of groups of psychologists doing similar things, putting in places things like, getting and emergency contact number so, whether it’s kind of somebody in the house or a relative if they’re living on their own, somebody I can contact in an emergency if they just disappeared, from a, from an online session. You haven’t got that moment where you can kind of, chase them through the door and kind of say “wait stop, let’s, let’s talk about this” if they’re really risky. Um, so, it’s just thinking about the safety stuff which I think overtime we kind of got a little bit more used to.”  P4: “we’ve put such a big emphasis is on, gosh I need to make this safe, because you’ve not got a person in the room, physically, with you, and it felt, risky? But, actually just unpicking what that riskiness, is, actually and kind of quantifying that and then putting in extra safety measures.” |
|  |  | 1.3. Anticipated therapeutic insufficiency | Therapists anticipated teletherapy would be limited for some modalities, tasks, and relational work, but direct experience often showed greater adaptability than expected. | P1: “The, erm, so, perhaps I should, this is going back a little bit, I was doing EMDR before, I haven’t done that online. and I don’t, and haven’t felt comfortable doing that online…”  P6: “Um, so, but in delivering therapy, so CBT fairly straight forward, but if I was do-you know, you couldn’t do neuropsychological testing online, or you couldn’t, you know, when filling in questionnaires, you know quite often I’d sit there and go through the questions with somebody, it’s not… I haven’t found it as easy to do online, and I don’t have an answer as to why that is, um, either. And something like EMDR or some other therapeutic modality where you, you kind of, need to do something more erm, practical with somebody, it doesn’t work as well.” (P6 has not done EMDR via teletherapy)  P1: “Erm, but I have done, trauma focused CBT, on here. And, found that that’s worked ok, so, I might just be finding barriers for myself.”  P3: “I suppose, in terms of differences in terms of therapeutic work that I do in compassion focused therapy, some of the work is called chair work, where I actually get people to move around from one chair to another and get into different emotional states in different chairs or talk to different parts of themselves, that’s’ harder online, because we’re just like this. So, there are some therapists that are working out who to adapt chair therapy to online, for example getting people to put on different hats or a different colour scarf or something.”  P5: “And erm, and so, my supervisor actually then told me, “actually I’ve been doing this for, for you know, years, and I’ve modified the way that I do my EMDR so rather than faffing about with, with apps on screen that some people watch a light kind of go from side to side, and just do tapping with people”. So, in fact I’ve got my pile of books in front of me here so I have a ton of books here and they do this as well and I just tap like this and they copy me, and, erm. So, at first, I was really, erm, I really struggled to see how that would work so effectively as the, you know, as light bar, but actually my supervisor did a session on me about something relatively innocuous, and erm, and, it was really so super effective I just thought wow, you know it was, it really surprised me.”  P5: “No matter what sort of treatment modality I’ve been working on, er you know, working on with them, and erm, yeah, I mean to be honest it’s been so much better than I thought it would be.” |
| 2. Context-Medium Differentiation | Participants described problems that initially seemed like problems with teletherapy but were later recognised as effects of working from home, lockdown conditions, domestic environments, or altered face-to-face conditions. | 2.1. Home-working and boundary disruption | Some difficulties arose because therapy was being conducted from home rather than because of teletherapy itself. | P1: “Because one of the, one of the big things that, theme I’m coming across, working at home, is boundaries between work time and finishing work and relax time and the blurring of those boundaries.”  P2: “Um, and I think it's, it's hard, there's pros and cons about it because also, needing to, make sure that your own boundaries are there, because you're kind of letting someone into your home, this isn't your normal therapy space.” |
|  |  | 2.2. Therapeutic setting changes | Participants reflected on how the changed setting, and not just the medium, shaped interaction, containment, and the feel of therapy. | P2: “Also, I think there's something about the therapeutic alliance where people quite like that [domestic setting]? Because they see you as being a person. So, if there's noises off or the doorbell rings or the dog suddenly manages to barge into the room, that in some ways helps the therapeutic alliance. At least it has with some of the clients I've seen.”  P2: “they just want you to be somebody who's in this same room all the time, nothing changes, it's not worked like that under lockdown. There's been too many things going on. So that has been a little bit harder.”  P3: “But, my regular clients know, you know, you have to, we say at the start, you need to be in a private room, this is your time, face a window so you’ve got good light on your face, don’t have distractions, turn your phone off. They know all that stuff, so it’s like a therapy room, we create a therapy room here online,” |
|  |  | 2.3. Privacy and household interruption | Privacy, childcare, interruptions, and domestic space shaped attendance and engagement in ways that were environmental rather than inherently technological. | P4: “I think the, the ones that I can think of that have young kids, that was really hard, erm. My worst attenders being someone who has four kids of school age, cause, there’s just, not enough privacy and when the schools were closed and the kids were home, just, there was just no way she had any physical space or mental space, to kind of do, do online therapy sessions, so there’s just been, there’s just interruptions and breaks all the time so perhaps the people that’ve done better are the people who have had somewhere to be, at home, and, been able to kind of create that space and, erm, have enough privacy.”  P6: “families where somebody’s got kids and, although I’ve said one of the downsides is you get interrupted and what have you, actually, being able to access therapy even though you’ve got disruption is better than having no therapy, at all.” |
|  |  | 2.4. Explicit differentiation of pandemic and medium | Participants explicitly distinguished pandemic-related barriers from teletherapy itself, and in some cases found altered in-person work more distancing than video sessions. | P1: “I think you know one of the, one of the, it’s a covid barrier I think, more than anything else, it’s kind of those, if you’re doing things where, working with social anxiety or those kinds of issues and you’re trying to kind of, you know, you want to kind of help practice skills within those settings or reflect on those settings and someone hasn’t been in a social situation for the last six months it’s kind of like you know, those are-But I think they’re kind of covid barriers rather than online therapy barriers.”  P6: “The other downside isn’t so much about the therapeutic interaction it’s actually having access to other people around, you know so, even though if I need professional supervision, I can access that, having an office where you’ve got people and you can just go and, make a cup of tea and a bi-, it, it makes it feel, as if you’re not alone. And I think as a therapist, to feel isolated, certainly I think has an impact on how you respond to people therapeutically as well. But I suppose, that’s, that’s a different thing you know, maybe, somebody is in an office doing, online therapy, that’s quite different, I’m talking a lot about being at home which is the pandemic situation really.”  P1: “Yeah well, so, where I, when this sort of obviously first erm, early stages, and I was seeing people face to face, the change in terms of masks and distance and the very kind of, I mean. It’s, that kind of, that felt harder, in terms of therapeutic alliance than on video interestingly.”  P4: “Erm, but yeah, even then, some of them, they’ve kind of, the kind, the kind that have retreated that said, actually lockdown suited them, and they’ve done quite well and have been able to focus on, focus on the tasks of therapy.” [“them” being clients with anxiety] |
| 3. Medium Affordances | Participants described teletherapy as a distinct therapeutic medium with its own material, perceptual, relational, and practical affordances. These were not solely deficits, but changes in how therapy could be conducted. | 3.1. Digital Resources and Technical Mediation | Teletherapy altered how therapists accessed, displayed, and used therapeutic materials, while also introducing medium-specific technical disruptions. | P1: “Erm, the sharing of materials initially was quite difficult, erm you know, if I wanted to kind of bring a diagram or whatever else, but that then subsequently kind of sussed out zoom, and the ability to do that there.”  P2: “Oh, and also, paper! Not being able to just write something down on a piece of paper in front of you and just kind of illustrate, like, draw a formulation. I've been having to make it on the screen and share your screen, that kind of thing has been a real pain, I have found that quite difficult.”  P2: “There are other things that kind of pros and cons, so, whereas before I would have to, kind of know that I've got everything set up and ready to go, any kind of scripts I might want to use, relaxation scripts of videos I might want to play, I have to kind of make sure they're available to me in the therapy room. And it can then be hard if you're online, but actually it's quite quick to have realised, that if I want to play a video about ACT then I can quickly bring it up on my screen and then share the screen.”  P6: “yeah and connectivity issues I find really difficult, I’ll quite often be in a situation where somebody has been very emotional, very upset, and at, my connectivity goes or theirs does, and you end up-they freeze, and, and you know, they have to keep repeating it and, that is awful. And that just doesn’t work at all, you know.” |
|  |  | 3.2. Reconfigured communicative and embodied cues | The medium changed what therapists could perceive and do interpersonally, including both losses in embodied presence and gains in facial visibility. | P2: “But, there’s things like, not being able to offer a glass of water or, pass somebody tissues, it makes that, sort of, the empathy a little harder, the lean in isn’t possible.”  P3: “I think touch is missing as well, I’m not a touchy therapist but just being able to shake somebody’s hand or sort of put a hand on their arm or your hand, if they’re upset that’s missing which I think is a bit of a shame.”  P1: “I think it’s the sensitivity to safety and sort of the wellbeing of the client being able to kind of, notice maybe, the finer cues, and be able to kind of discuss those and kind of, I suppose for me, it’s about, erm, yeah I think that, that’s the primary thing, working with those kind of issues I suppose.”  P6: “So, say if somebody, talking about something that makes them feel anxious, quite often, they’ll have a tell, so something like their leg shaking or tapping their foot or, you know do, well see I do this with my hands. But somebody might be doing something off screen that as a clinician I would want to pick up on because it might be an important indicator you know, about, what they might be talking about and the impact that actually has on them, as well.”  P5: “you do get quite a close-up view of people’s faces and their facial expressions and so I think sometimes, there’s less to be missed in terms of, picking up something expressive that somebody’s doing with their eyes or their face, erm, in some kind of way.” |
|  |  | 3.3. Intensified attentional demands | Therapists described teletherapy as unusually effortful and intense, requiring sustained visual and interpersonal focus. | P1: “Then there was this kind of bit about, actually this is quite tiring for people. So, whilst I’ve got used to it, then a new client comes along, who isn’t perhaps doing therapy online, they’re then, pract-you know, got to be aware that they may be experiencing these other things.”  P4: “But it is, it’s sort of, it’s all, it’s all here isn’t it, the focus rather than having a sense of the person in the room with you. So, it’s just it can feel, it can feel more intense and clients can feel a little uncomfortable with that level of intensity, I think, so there’s a lot to, to try and read, in a different way.”  P6: “And, you know, to some extent, in some sense there’s less distractions because you’re just focused on them as opposed to being sat with somebody and maybe, I don’t know. Because in, in person social communication you tend to take in more surroundings, don’t you? Whereas on something, I think, like zoom or teams, you’re kind of focused on the people you’re talking to more, so. Which makes it more exhausting as well, actually, erm, yeah.” |
|  |  | 3.4. Accessibility, flexibility, and autonomy | Teletherapy was experienced as increasing accessibility, reducing logistical barriers, and expanding client choice and control. | P1: “And, kind of, upsides and think that just the, the, breakdown of the barriers of location, erm, so, in terms of people just being able to access, therapists more easily in that way, they’ve got the technology.”  P3: “it makes it more accessible for people with mobility issues or anxiety issues”  P4: “benefits, the first things that come to mind are greater accessibility, erm, it’s a, it’s a hell of a commitment for people to commit to weekly therapy if they’re um, coming after work or in their lunch hour or kind of fitting it around family commitments. Generally, I mean people have been traveling or adding on quite a bit to their day to attend a therapy appointment, I think that, yeah, the greater accessibility of, fitting it in somewhere. It’s, it becomes an hour a week instead of two or even three hours a week, you know including the traveling, so I think that, in terms of their weekly routine and their timings, and maybe being able to slot stuff into their life, they’ve got greater choice over where they may be able to put that now.”  P4: “Actually the, I’m seeing clients from all over the place now, I mean, well not all over, but there’s kind of, much bigger geographical spread actually. Um, so that’s, that’s, I think that’s kind of one of the nicer differences, it just opens, opens up choice for clients. Erm, and they can kind of, they really do have a much wider, um, pool of therapists to pick from.”  P6: “Erm, they can sort of switch the computer on when they want, they don’t have to wait for somebody to come and knock on the door, which can sometimes be a stressful thing, you know, erm. I make sure that the people I work with have control so they send me the invite as opposed to me inviting them in, because it gives them that sense of power over a session, then. So, no I think I feel, they like the fact they’re approaching me and that’s in their control.” |
|  |  | 3.5. Differential client-medium fit | Therapists observed that the medium appeared to suit some clients particularly well, suggesting that teletherapy interacted differently with different client characteristics and life circumstances. | P1: “I have seen a lot more IT people, and I think that perhaps, as a population they quite often find it difficult to seek help, and engage talk to people, privately.”  P1: “So, it’s attracted some people that have, so, I see quite a lot of university students, they, they’ve been able to do this because, they can be going home and back to uni and whatever and it can all remain consistent, so we can sort of do the same time same place that they’re, it’s online so.”  P6: “So, people with autistic spectrum disorders for example, informal feedback tells me that they actually find it [therapy] easier online.” |
| 4. Relational Viability and Selective Integration | Therapists increasingly came to view teletherapy as a viable, though distinct, relational format. They described therapeutic relationships as achievable online, but evaluated teletherapy’s longer term role selectively rather than universally. | 4.1. Alliance preserved but reconfigured | Therapists came to see the therapeutic alliance as achievable online, although sometimes altered in texture, closeness, or cues. | P1: “I thought it would be, erm, diffic-more difficult, or, well, more difficult than it is to develop a relationship or to form a therapeutic alliance. Erm, and to, I suppose, perhaps read some of that, how much it mattered to be able to read some of those finer cues within the session. Erm, and I think, erm, yeah, having moved forward into this way of working I think that kind of manifests itself anyway, without you having to be in the same room.”  P5: “Because I think, erm, I’d say the same things, I make people comfortable in the same kind of ways, erm, and the, and the way that I respond to people is based on their response to me. So, erm, other than the fact that I can’t see their full body language you know, and in terms of them telling me one thing and then doing something else, which I don’t think has been an issue at all, I don’t think there’s anything about doing it through zoom, I mean I’ve been using zoom mostly, that has really interfered with the um, process at all of developing a relationship with the, with the client.”  P3: “it’s a different kind of relationship, erm. Yeah, I’d say it’s just different, I still think there’s good relationships being built and it still feels trusting it’s just, different. I guess it’s like, meeting somebody in a room versus meeting them in a park, you know, it’s just different.”  P6: “Erm, in person, you are, in my mind, you are able to develop a more close relationship. You, you get to see that person if they’re not in clinic, you get to see them in their own surroundings, you can pick up on thing that you don’t pick up on when you’re looking at the across a screen…”  P5: “At first, I was quite anxious about doing it I was quite worried about, not being able to make the connection with people, not being able to, erm, you know, not be able to read people in some kind of way, but actually the, I’ve been proved completely wrong, I’ve found the opposite has been true for me personally.” |
|  |  | 4.2. Evaluating fit and sustaining integration | Participants increasingly evaluated teletherapy as effective and worth retaining, but in differentiated ways depending on client, therapist, and therapeutic task. | P2: “Yeah, probably, because I think at the beginning it thought it was going to be much harder than it actually was, I think we all did. I think we all thought that any of this online stuff was just going to be, “nah we don't do that it's too difficult”, and it's been a lot better than I thought it was going to be.”  P1: “Erm, but I think, I think, every client is going to find it different like every therapist is going to find it different, and I think just over time I found different clients over time have experience it differently as well.”  P4: “The, they’ll always benefit from that, it’s an, I think they’ll run nicely side by side, I’ve dropped the work and I’m going to keep an online clinic actually. So I’m going to, when I do get back to my rooms, I’m going to have an in-person clinic and an online clinic, that’s my plan. Erm, so, kind of keep a, keep a bit of mixture of both so I see them, I see the benefits of both. Erm, and yeah, I think it’s important to, for people to have a choice, so people can test it out and if, actually if online doesn’t work then they might need face to face, it’s kind of just throws a couple of factors up, I think.”  P3: “Definitely yes, I’m considering staying online in fact. Which I think is really interesting, I might change my mind, six months down the line I might say “I just need to get out”.”  P6: “You know as access to psychological therapies for the general population, erm, in general, then absolutely I think, there are ways that teletherapy could replace many of the services that are overwhelmed and you know just aren’t working as efficiently as they could. Will it replace all types of psychological input or therapy services, I don’t think so, I don’t think it can.”  P3: “But, we’re still fine as we can develop a really good therapeutic relationship, and people are getting better. So, it’s working.”  P5: “But I think, erm, there are probably positives and negatives but from my point of view I see quite a lot of positives really, and I would prefer, I think to continue to make it work.” |
